# Lactulose Increases Equol Production and Improves Liver Antioxidant Status in Barrows Treated with Daidzein

**DOI:** 10.1371/journal.pone.0093163

**Published:** 2014-03-25

**Authors:** Weijiang Zheng, Yanjun Hou, Wen Yao

**Affiliations:** 1 College of Animal Science and Technology, Nanjing Agricultural University, Nanjing, China; 2 Key Lab of Animal Physiology and Biochemistry, Ministry of Agriculture, Nanjing, Jiangsu, China; Oklahoma State University, United States of America

## Abstract

Equol, one of the intestinal microflora metabolites of daidzein, has gained much attention for having greater bioactivity than its precursor (daidzein and daidzin) and seeming to be promoted by hydrogen gas. The effects of lactulose on the equol-producing capacity and liver antioxidant status of barrows treated with daidzein were investigated in this study. Male castrated piglets (barrows) of Landrace×Duroc, aged 40 days, were randomly divided into the following three groups: control group (C, n = 12, fed an isoflavones-free basic diet), daidzein group (D, n = 12, fed an isoflavones-free basic diet with 50 mg/kg of daidzein supplementation) and daidzein+lactulose group (D+L, n = 12, fed an isoflavones-free basic diet with 1% of lactulose and 50 mg/kg of daidzein supplementation). After 20 days, the profile of short-chain fatty acids in the colon digesta showed that lactulose significantly increased the fermented capacity in the gastrointestinal tract of the barrows. First-void urinary equol concentrations were significantly higher in the D+L group than in the D group (3.13±0.93 compared to 2.11±0.82 μg/ml, respectively). Furthermore, fecal equol levels were also significantly higher in the D+L group than in the D group (12.00±2.68 compared to 10.00±2.26 μg/g, respectively). The population of bacteroidetes and the percentage of bacteroidetes to bacteria in feces were higher in the D+L group than in the D group. The DGGE profiles results indicate that lactulose might shift the pathways of hydrogen utilization, and changing the profiles of SRB in feces. Moreover, the D+L group had weak enhancement of T-SOD and CuZn-SOD activities in the livers of barrows treated with daidzein.

## Introduction

Daidzein, the main isoflavones found in soybeans and most soy foods, can be transformed to equol [1]. Equol is exclusively produced by intestinal microflora [2] and may be superior to all other isoflavones in terms of its antioxidant [3] and anti-inflammatory properties [4]. Among humans, 30–50% has the intestinal bacteria capable of producing equol [5]. Equol producers are advantageous in terms of enhancing health benefits, and gained much attention since the postulation of the equol hypothesis [6]. Although there is conflicting evidence available regarding the potential health benefits associated with the ability to produce equol, there is a growing interest in dietary applications that might enhance equol production in humans and animals.

Hydrogen gas was reported to stimulate equol production in an equol-producing mixed culture (EPC4), likely by acting as an electron donor in the formation of equol [7]. In the presence of daidzein, EPC4 significantly decreased the methanogenesis and sulphidogenesis in the cultures of faecal samples with methanogenic or sulphate-reducing abilities [8]. A stronger decrease was observed with increasing amounts of EPC4 and constant equol production, suggesting daidzein to act partly as a hydrogen sink.

Lactulose, a disaccharide formed from one molecule each of the simple sugars fructose and galactose, cannot be digested and absorbed by the body, but can be digested by bacteria colonizing the gastrointestinal tract, especially the colon. Oral administration of lactulose can significantly increase breath H_2_ production [9]. We hypothesized that equol production could be regulated by adding a hydrogen-producing prebiotic *in vivo*/*in vitro*. So far, no relationship between a hydrogen-producing prebiotic and equol production has been studied *in vivo*. The current study was designed to examine the effects of lactulose on the equol production and liver antioxidant status of castrated male piglets treated with daidzein.

## Materials and Methods

### Statement of Ethics

Animal care and use were conducted in accordance with the Animal Research Institute Committee guidelines of Nanjing Agricultural University, China. The study’s castrated male piglets (barrows), aged 40 days and initially weighing 9.35±0.17 kg, were crossed from Landrace and Duroc (weaned at 28±1d of age). All pigs were housed in an environment-controlled room and maintained under the care of the Laboratory Animal Unit from the Institute of Experimental Animal Center, Jiangsu Academy of Agricultural Sciences, China. Within the house, each of the six pigs were housed in a pen (4×4 m) with one feeder and two nipple drinkers to allow them ab libitum access to feed and water. This study was approved by the Committee of Animal Research Institute, Nanjing Agricultural University, China.

### Animal Husbandry, Diets, Experimental Design, and Sampling

Barrows were given free access to an isoflavones-free diet for five days before the feeding trial and then randomly divided into the following three groups: control (C, n = 12, fed an isoflavones-free basic diet), daidzein (D, n = 12, fed an isoflavones-free basic diet with 50 mg/kg of daidzein supplementation), and daidzein+lactulose (D+L, n = 12, fed an isoflavones-free basic diet with 1% of lactulose- and 50 mg/kg of daidzein supplementation). Table S1 shows the composition and nutrient levels of the experimental diets, which were prepared according to the nutrient requirements [10] for weaned pigs.

After 20 days of the feeding trial, first-void urine and fecal samples were collected and stored at −70*°*C until assay. On the 22nd day, six pigs from each treatment were randomly selected and killed humanely by an intramuscular injection of sodium pentobarbital (40 mg/kg body weight). The liver, colon mucosa, and digesta were sampled and stored at −70*°*C until assay.

### Extraction and Quantification of Daidzein and Equol

Urine samples were prepared as previously described [11]. In brief, each 2 ml of centrifuged urine sample (centrifuged at 850 g for 15 minutes) was mixed with 7 ml of sodium acetate buffer (pH 5.0) and 30 μl β-glucuronidase (GUS) from *Escherichia coli*(100 U/ml)/30 μl Sulfatate from *Helix pomatia* (500 U/ml) and incubated for three hours at 37*°*C, followed by extraction with 7 ml of ethyl acetate. The solvent was removed under a gentle stream of nitrogen, and the sample was re-dissolved in 500 μl 80% (V/V) methanol prior to analysis.

Faecal daidzein and equol were analyzed according to a method previously mentioned [12]. Faecal samples (1 g) were extracted with 7 ml of ethyl acetate. The solvent was removed under a gentle stream of nitrogen, and the sample was re-dissolved in 3 ml 80% (V/V) methanol prior to analysis.

Concentrations of daidzein and equol were determined using a HPLC system consisting of a Surveyor MS Pump Plus, Surveyor Autosampler, Surveyor PDA Plus (Thermo Fisher Scientific, Finnigan™, USA), and the Thermo Fisher Xcalibur™ software package (version 1.4 SR1). A 20 μl sample was injected and separated over a YMC-Pack Pro C18 column with a particle size of 5 μm (YMC Inc., Japan) kept at 30°C. Elution was isocratic with a mobile phase consisting of 20% acetonitrile, 30% methanol, and 45% water and the flow rate was maintained at 300 μl/min. The eluate was analyzed with Surveyor photodiode array (PDA) Plus detection system (Thermo Fisher Scientific, Finnigan™, USA), UV spectra of the peaks for equol was detected at 205 nm, and daidzein was detected at 260 nm. UV-absorption spectra allowed identification of the peaks after comparison with pure standards. Calibration curves for quantification of daidzein and equol were constructed using pure standards obtained from Sigma (New York). The concentration of equol was calculated following the equation Y = 85653.6+1.23123e+006*X (y: HPLC peak area; x: concentration of equol) [13]. The concentration of daidzein was calculated following the equation Y = 36529.5+844385*X (y: HPLC peak area; x: concentration of daidzein) [13].

### DNA Isolation, PCR Amplification, and DGGE Analysis

A QIAamp® DNA Stool Mini Kit was purchased from QIAGEN Germany and used to extract DNA from the fecal, colon digesta, and mucosa samples according to the manufacturer’s instructions. DNA extracts were stored at −20°C until ready for use.

Primers U968-GC and L1401 [14] were used to amplify the V6–V8 regions of the bacterial 16 S rRNA gene. Primers 519f and 915rGC [15] were used to amplify the 16S rDNA of methanogenic *Archaea*. Primers APS-FW and APS-RV-GC [16] were used to amplify a 436-bp fragment of the adenosine-5′-phosphosulfate (APS) reductase subunit A gene. The primers used in this study are listed in Table S2. Oligonucleotide primers were ordered from Invitrogen (Shanghai, China). PCR amplification was performed in a T1 Thermal Cycler (Biometra, Germany). PCR products (5 μl) were analyzed by electrophoresis on 1.5% agarose gel (w/v) to check the sizes and amounts of the amplicons.

PCR products of the total bacteria, methanogenic *Archaea,* and sulfate-reducing bacteria were separated by DGGE [17] using a Dcode™ System (Bio-Rad, Hercules, CA) in 8%, 6%, and 8% polyacrylamide gels with a denaturing gradient of 38–55%, 30–60%, and 32–65% (a 100% denaturant is defined as 7 M urea and 40% (v/v) deionized-formamide)respectively. The gels were run in a 0.5×TAE buffer. The electrophoresis was performed at 60*°*C for 10 minutes at a voltage of 200 V, and subsequently run at a fixed voltage of 85 V for 12 h at 60*°*C (with methanogenic *Archaea* for 16 h). After completion of electrophoresis, the gels were stained with silver nitrate (AgNO_3)_ [18] and scanned by a GS-800 Calibrated Densitometer (Bio-Rad) for analysis with GelCompare II 5.0 (Applied Maths, Kortrijk, Belgium).

### Real-time PCR Assay on the Number of Selected Bacteria

Firmicutes [19], bacteroidetes [20], [21], *methanogen* (mcrA) [22], and subunit A of the adenosine-5′-phosphosulfate (APS) reductase gene [16] from our previous experimental DNA samples (unpublished data) were amplified with the primers shown in Table S2. Purified PCR products were cloned in *Escherichia coli* JM109 using the pGEM-T vector system (Invitrogen, Shanghai, China). Plasmid DNA with correct size inserts was extracted and used for constructing standard curves. The standard curves were generated using triplicate ten-fold dilutions of plasmid DNA. For all real-time PCR assays, there was a linear relationship between the log of the plasmid DNA copy number and the calculated C_T_ value across the specified concentration range (R^2^ = 0.99 in all cases) (data not shown). Real time PCR was carried out using an ABI 7300 real-time PCR machine (Applied Biosystems, Foster City, CA, USA) in a total volume of 20 μl containing 10 μl 2 × SYBR Premix Ex Taq (Takara Dalian China), 2 μl DNA (1∶9 diluted with PCR-grade H_2_O), 0.4 μl each of 10 μmol/L forward and reverse primers, 0.4 μl ROX, and 4.2 μl PCR-grade water. The program was set at 95°C for 10 seconds, followed by 40 cycles of 95°C for 5 seconds, and 60°C for 31 seconds. The melting curve analysis of amplification products was performed at the end of each PCR reaction to confirm that only one PCR product was amplified and detected.

### Biochemical Analysis of Oxidative and Anti-oxidative Biomarkers in Liver, and the Biochemical Analysis of SCFA in Colonic Digesta

The antioxidant assays were conducted using assay kits purchased from the Nanjing Jiancheng Institute of Bioengineering (Nanjing, Jiangsu, China). One hundred milligrams of frozen liver tissue in 1 ml of homogenization buffer (0.9% cool physiological saline) was homogenized on ice with a Polytron-aggregate homogenizer (POLYTRON PT-1200E, Lucerne, Switzerland) for 30 seconds at 12500 rpm. The homogenate was centrifuged at 2500 rpm for 10 minutes at 4°C, and the resultant supernatant was a 10% concentration. In following, 0.9% of physiological saline was used to dilute the supernatant to different concentrations, which were then stored at −20*°*C until analysis. All following assays were conducted according to the manufacture’s procedures.

The VFAs in the colon digesta were measured by using a capillary column gas chromatograph (GC-14A; Shimadzu, Japan) according to Mao et.al [23].

### Calculations and Statistical Analysis

DGGE analysis of all samples was repeated twice. All gels were scanned at 400 dpi. The number of DGGE bands and similarity indices were calculated from the densitometric curves of the scanned DGGE profiles using the GelCompare II 5.0 software (Applied Maths, Kortrijk, Belgium) with the Pearson product-moment correlation coefficient [24]. Similarity indices were calculated for pairs of DGGE profiles for the colon mucosa, digesta, and feces samples, respectively. As a parameter for the structural diversity of the microbial community, the Shannon index of general diversity, H′ [25], was calculated [26].

All statistical analyses were performed using a one-way ANOVA with SPSS statistical software (ver. 18.0 for Windows, SPSS Inc., Chicago, IL) The differences were considered significant at P<0.05. Differences among treatments for parameters were tested for significance using Duncan's test. Data are presented as means±SD.

## Results

### SCFA Profile in the Colon Digesta

Table 1 shows the effect of lactulose and daidzein on the SCFA profile in colon digesta. Although little difference was found between the D and C groups, large effects of lactulose were found. The acetic acid, propionic acid, butyric acid, and valeric acid concentrations in the colon digesta between the D and D+L groups showed no significant difference (Table 1). However, isobutyric acid, isovaleric acid, and branched chain fatty acid (BCFA), as well as the total VFA levels in the colon digesta, were significantly higher in the D+L group than in the D and C groups (Table 1).

**Table 1:** Effects of lactulose on SCFA levels in the colonic digesta of barrows fed with daidzein.

Items	Control(n = 6)	Daidzein(n = 5)	D+L(n = 6)	P
Acetic acid (mM)	31.39±5.11	31.69±3.51	36.44±2.56	0.069
Propionic acid (mM)	10.39±0.75	11.35±1.41	11.86±1.70	0.191
Isobutyric acid (mM)	1.17±0.35^b^	1.25±0.22^b^	1.80±0.40^a^	0.010
Butyric acid (mM)	4.07±0.63^b^	4.98±0.63^ab^	5.44±1.03^a^	0.025
Isovaleric acid (mM)	1.76±0.18^b^	1.87±0.58^b^	2.66±0.30^a^	0.002
Valeric acid (mM)	1.18±0.14^b^	1.53±0.34^a^	1.56±0.19^a^	0.025
BCFA (mM)	2.93±0.40^b^	3.12±0.77^b^	4.46±0.67^a^	0.001
TVFA (mM)	49.96±6.16^b^	52.66±4.66^b^	59.77±4.38^a^	0.013

Note: values with different small letter superscripts in the same row mean that there was a significant difference (*P*<0.05), and the same small letter superscripts mean that there was no difference.

### Levels of Daidzein and Equol in the Urine and Feces

Thirty-two individuals completed urine and feces sample collection (from 10 pigs in C group, 11 in D group, and 11 in the D+L group, respectively). The concentrations of daidzein and equol in the urine and feces in the barrows treated with daidzein were significantly higher than those in the control pigs (Table 2). The urinary equol concentrations and the ratio of equol to daidzein were significantly higher in the D+L group than in the D group. However, no significant difference was observed in daidzein concentration in the urine and feces between the D and D+L groups. Fecal equol levels were significantly higher in the D+L group compared to the D group (Table 2). No difference was found in the ratio of fecal equol to daidzein concentration between the D and D+L groups (Table 2).

**Table 2:** Effects of lactulose on the concentrations of urinary and fecal daidzein and equol, and the ratio of urinary and fecal equol to daidzein concentrations in barrows fed with daidzein.

Items	Control	Daidzein	D+L	P
	(n = 10)	(n = 11)	(n = 11)	
Urine				
Daidzein (μg/ml)	ND^b^	9.70±2.69^a^	8.49±2.18^a^	<0.0001
Equol (μg/ml)	ND^c^	2.11±0.82^b^	3.13±0.93^a^	<0.0001
Equol to daidzein ratio	ND^b^	0.21±0.06^b^	0.38±0.10^a^	<0.0001
Faecal				
Faecal daidzein (μg/g)	ND^b^	3.92±1.48^a^	3.84±1.93^a^	<0.0001
Faecal equol (μg/g)	ND^c^	10.00±2.26^b^	12.00±2.68^a^	<0.0001
Equol to daidzein ratio	ND^b^	2.90±1.40^a^	3.77±1.57^a^	<0.0001

Note: values with different small letter superscripts in the same row mean that there was a significant difference (*P*<0.05), and the same small letter superscripts mean that there was no difference. ND = not detected.

### Microflora Profiles of Total Bacteria, Methanogenic Archaea, and Sulfate-reducing Bacteria (SRB) in Feces, Colon Digesta, and Mucosa

**Figure 1:**
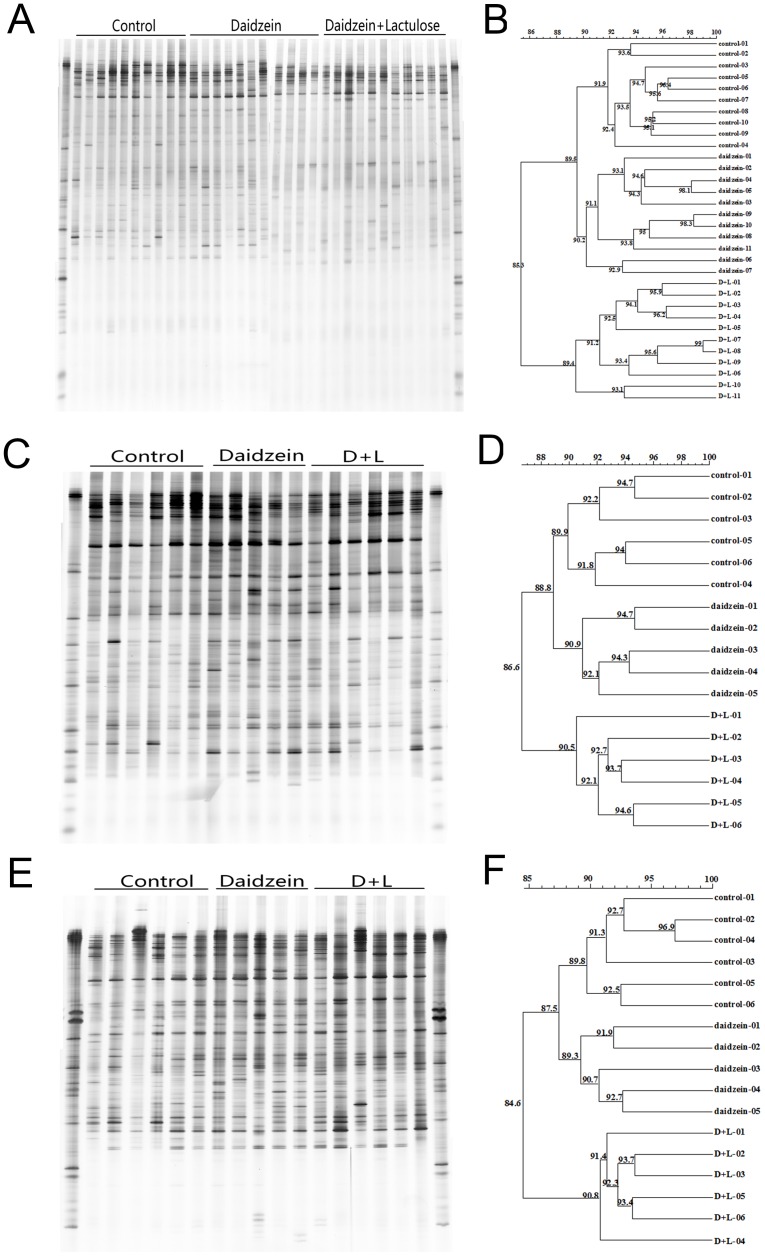
DGGE profiles and similarities of bacterial communities in the feces (A)(B), colonic digesta (C)(D), and colonic mucosa (E)(F).

**Figure 2:**
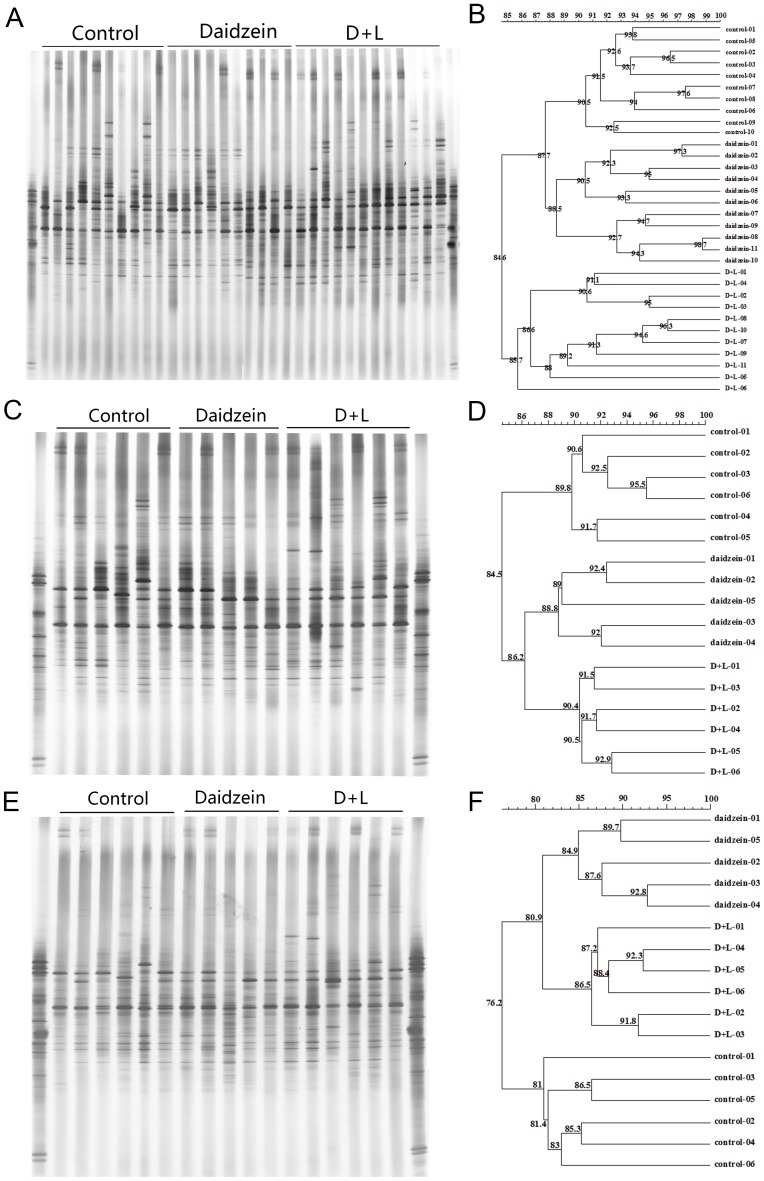
DGGE profiles and similarities of methanogenic *Archaea* in the feces (A)(B), colonic digesta (C)(D), and colonic mucosa (E)(F).

**Figure 3:**
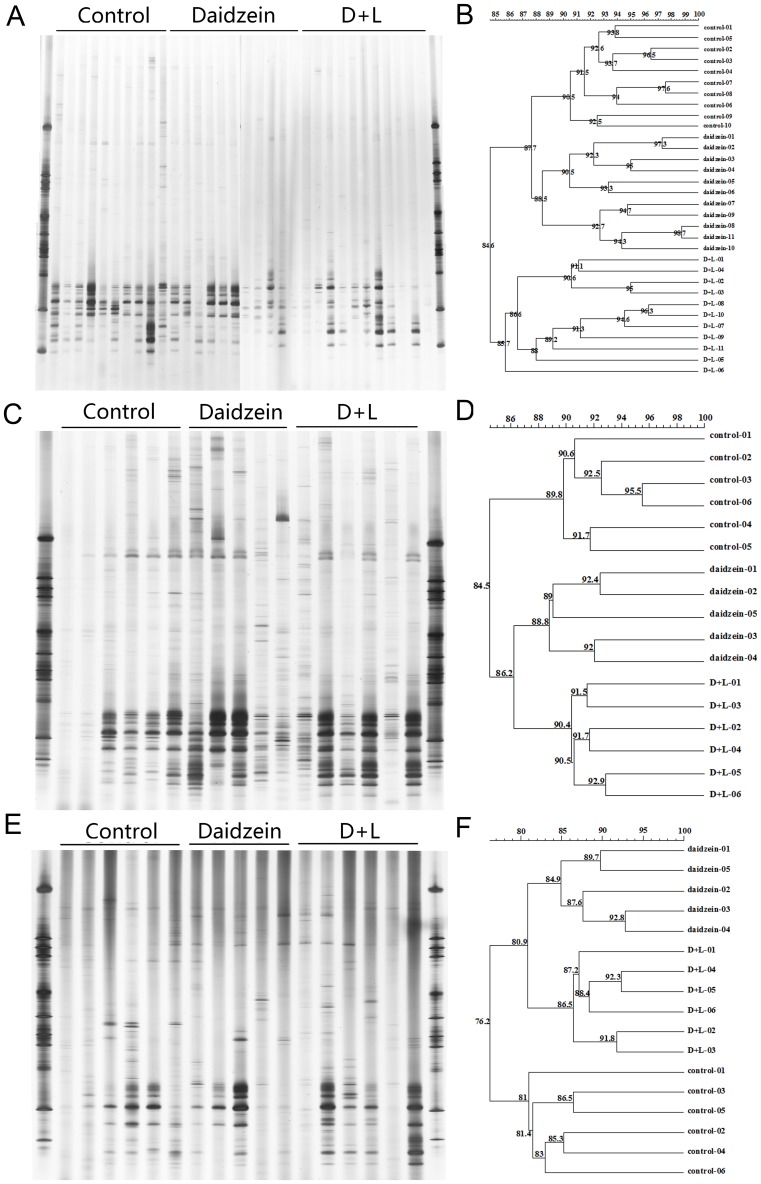
DGGE profiles and similarities of APS reductase subunit A gene in the feces (A)(B), colonic digesta (C)(D), and colonic mucosa (E)(F).

The representative DGGE analysis of the PCR fragment generated with primers U968GC and L1401, 519f and 915rGC, and APS-FW and APS-RV-GC are shown in Figure 1 (A, C, and E), Figure 2 (A, C, and E), and Figure 3 (A, C, and E), respectively. For the total bacteria DGGE profile, the predominant bands in the fecal DNA samples were on the upper gels (Figure 1 A), whereas in the colon digesta and mucosa, the predominant bands were evenly separated in the gels (Figure 1 C and E). Although some bands changed for different groups, no particular bands were commonly changed by dietary treatment. Cluster analysis revealed that the overall similarity of bacteria DGGE profiles in feces, colon digesta, and mucosa were 85.3%, 86.6%, and 84.6%, respectively (Figure 1 B, D, and F). Interestingly, in three different sites the C and D groups were all in the same cluster with a similarity of 89.5%, 88.8%, and 87.5%, respectively (Figure 1 B, D, and F).

For the methanogenic *Archaea* composition in the fecal, colon digesta, and mucosa of the barrows, the predominant bands were all in the middle of the gels (Figure 2 B, D and F). There were no special bands influenced by daidzein or the D+L treatment. Cluster analysis revealed that the overall similarity of the MPB DGGE profiles in feces, colon digesta, and mucosa were 84.6%, 84.5%, and 76.2%,respectively (Figure 2 B, D, and F). The C and D samples were found in the coherent cluster with a similarity of 87.7% in the feces of the barrows. However, the D and D+L samples were found in one cluster with a similarity of 86.2% and 80.9% respectively in the colon digesta and mucosa samples of the barrows (Figure 2 B, D, and F).

For the DGGE analysis of the APS reductase DNA amplicons in three different locations, the bands number seemed less than the number of MPB and bacteria. The predominant bands were all at the bottom of the DGGE gels. Still, no particular bands were found to be influenced by daidzein or the D+L treatment. Cluster analysis showed that the overall similarity of the APS reductase DGGE profiles in feces, colon digesta, and mucosa were 50.4%, 69.6%, and 67.7%, respectively. The C and D groups were in the same cluster with a similarity of 55.8% in the feces samples. With the DNA samples from the colon digesta, the D and D+L groups were in one cluster with a similarity of 77.1%. Lastly, the cluster analysis for the colon mucosa in the C and D+L groups were in the same cluster with a similarity of 71.9% (Figure 3 B, D, and F).

To further examine the effects of lactulose and daidzein, the number of predominant bands in the DGGE profiles and their Shannon index of general diversity were analyzed (Table 3). For the fecal samples, major effects were found in the DGGE bands for bacteria, MPB, and SRB, while no differences were found in the Shannon diversity of selected bacteria. The band number of the DGGE gels for bacteria was significantly lower in the D+L group than the C and D groups, and the band number of the DGGE gels for MPB in the C and D+L groups was significantly higher than in the D group. The DGGE band number for SRB in the D+L group was significantly lower than in the C and D groups, and the C group had a higher DGGE band number for SRB than in the D group (Table 3).

**Table 3:** Effects of lactulose on the number of DGGE bands and Shannon diversity of the feces and colonic samples of pigs fed with daidzein.

Target group	DNA sample	Items	Control	Daidzein	D+L	P
Total bacteria	Feces^A^	DGGE bands number	57.80±3.52^a^	57.81±3.87^a^	51.00±2.32^b^	<0.0001
		Shannon Diversity	3.53±0.15	3.55±0.16	3.42±0.11	0.095
	Colon digesta^B^	DGGE bands number	76.00±2.61^a^	77.40±2.88^a^	69.50±4.32^b^	0.003
		Shannon Diversity	3.71±0.28	3.94±0.08	3.81±0.16	0.200
	Colon mucosa^C^	DGGE bands number	67.17±5.85^ab^	71.20±3.35^a^	63.17±2.22^b^	0.021
		Shannon Diversity	3.81±0.16	3.91±0.04	3.82±0.11	0.350
MPB	Feces^A^	DGGE bands number	41.40±1.58^a^	38.55±1.64^b^	41.18±1.66^a^	<0.0001
		Shannon Diversity	3.07±0.24	3.09±0.16	3.21±0.15	0.164
	Colon digesta^B^	DGGE bands number	55.33±1.75	57.20±3.42	58.33±2.58	0.170
		Shannon Diversity	3.32±0.26	3.32±0.11	3.26±0.19	0.827
	Colon mucosa^C^	DGGE bands number	35.67±4.84^b^	43.80±4.82^a^	44.83±2.48^a^	0.004
		Shannon Diversity	3.16±0.22	3.25±0.27	3.34±0.14	0.333
SRB	Feces^A^	DGGE bands number	42.40±7.44^a^	30.00±7.95^b^	23.45±5.65^c^	<0.0001
		Shannon Diversity	2.58±0.26^a^	2.34±0.28^ab^	2.20±0.35^b^	0.031
	Colon digesta^B^	DGGE bands number	55.83±12.19^b^	78.20±3.96^a^	67.67±6.31^a^	0.002
		Shannon Diversity	3.26±0.25	3.52±0.16	3.28±0.33	0.237
	Colon mucosa^C^	DGGE bands number	50.50±6.67^a^	39.20±3.90^b^	49.83±4.54^a^	0.006
		Shannon Diversity	3.02±0.28	2.80±0.23	3.01±0.29	0.353

Note: values with different small letter superscripts in the same row mean that there was a significant difference (*P*<0.05), and the same small letter superscripts mean that there was no difference.

A The fecal sample replications in C group n = 10, D group n = 11, D+L group n = 11.

B The colon content sample replications in C group n = 6, D group n = 5, D+L group n = 6.

C The mucosa of colon sample replications in C group n = 6, D group n = 5, D+L group n = 6.

For the colon digesta samples, the results showed that there was no influence on the Shannon diversity of the DGGE profile for bacteria, MPB, and SRB, and that the DGGE band numbers for bacteria and SRB were of significant difference (Table 3). The band numbers of the DGGE profile for bacteria in the D+L groups were significantly lower than in the C and D groups, and the DGGE band numbers for SRB in the D and D+L groups were significantly lower than in the C group (Table 3).

There was no difference in the Shannon diversity of the DGGE profile for bacteria, MPB, and SRB in the samples of colon mucosa. However, the DGGE band numbers for bacteria were found to be significantly lower in the D+L group than the in the D group (Table 3). The DGGE band numbers for the MPB in the C group were significantly higher lower than the D and D+L groups, whereas no difference was found between the D and D+L groups. The DGGE band numbers for SRB in the D group were significantly lower than in the C and D+L groups (Table 3).

### Enumeration of Selected Bacteria in Feces, Colon Digesta, and Mucosa

To explore the further influence of lactulose and daidzein on the microorganisms, real-time RCR was used to quantify the abundance of total bacteria, firmicutes, bacteroidetes, methanogen-producing bacteria, and sulfate-reducing bacteria in the fecal, colon digesta, and mucosa. Table 4 shows the population and abundance of selected bacteria in the three different locations of the C, D, and D+L groups. For the fecal DNA samples, the number of total bacteria, firmicutes, and methanogen-producing bacteria in the three groups there was no difference. However, the population of bacteroidetes and sulfate-reducing bacteria in the D+L group was significantly higher than in the C and D groups. No difference was found between the C and D groups (Table 4). Still, the percentage of firmicutes and bacteroidetes to total bacteria was found to be significantly higher in the D+L group than in the C and D groups. Unexpectedly, excluding the sulfate-reducing bacteria in the mucosa of the colon, a significant difference was not found in the population and abundance of selected bacteria in the mucosa of the colon, or in the digesta of the colon in the barrows (Table 4). The population of sulfate-reducing bacteria in the mucosa of the colon was found to be significantly higher in C group than in the D and D+L groups (Table 4).

**Table 4:** The abundance of selected bacteria in the feces, colonic digesta, and mucosa of barrows.

DNA sample	Items	Control	Daidzein	D+L	P
Faecal^A^	Bacteria	(5.38±2.86)×10^11^	(5.10±1.50)×10^11^	(6.01±1.44)×10^11^	0.56
	Firmicutes	(3.24±1.68)×10^11^	(3.17±0.92)×10^11^	(4.18±1.16)×10^11^	0.133
	Bacteroidetes	(3.86±1.68)×10^10b^	(4.02±1.83)×10^10b^	(7.09±1.77)×10^10a^	<0.0001
	Firmicutes%	61.10±5.97^b^	62.15±4.46^b^	69.30±5.39^a^	0.002
	Bacteroidetes%	7.74±1.54^b^	7.89±3.21^b^	11.86±1.43^a^	<0.0001
	Methanogen	(4.06±0.91)×10^6^	(4.43±2.32)×10^6^	(3.57±2.67)×10^6^	0.641
	SRB	(4.89±1.49)×10^4a^	(3.68±0.64)×10^4b^	(2.71±1.12)×10^3c^	<0.0001
Colon digesta^B^	Bacteria	(3.88±2.75)×10^10^	(3.90±1.17)×10^10^	(4.07±2.32)×10^10^	0.9879
	Firmicutes	(2.45±1.68)×10^10^	(2.47±0.65)×10^10^	(2.67±1.60)×10^10^	0.9589
	Bacteroidetes	(5.75±2.86)×10^9^	(6.74±0.50)×10^9^	(7.54±3.18)×10^9^	0.4974
	Firmicutes%	63.62±3.93	63.85±2.75	65.19±3.25	0.6996
	Bacteroidetes%	16.13±3.41	18.20±3.90	19.71±4.01	0.2878
	MPB	(5.24±2.48)×10^6^	(3.57±1.99)×10^6^	(5.35±3.23)×10^6^	0.493
	SRB	(2.80±2.36)×10^4^	(3.98±1.30)×10^4^	(8.87±7.04)×10^4^	0.06
Colon mucosa^C^	Bacteria	(5.24±2.36)×10^8^	(4.91±1.78)×10^8^	(5.05±1.75)×10^8^	0.9636
	Firmicutes	(3.35±1.71)×10^8^	(3.09±1.12)×10^8^	(3.18±1.13)×10^8^	0.9498
	Bacteroidetes	(9.42±5.63)×10^7^	(8.81±2.27)×10^7^	(9.70±3.09)×10^7^	0.9353
	Firmicutes%	62.72±5.72	62.82±5.32	62.67±1.78	0.9983
	Bacteroidetes%	17.64±4.04	18.45±2.60	19.45±2.07	0.5906
	MPB	(4.50±3.00)×10^5^	(2.19±1.20×10^5^	(3.10±2.06)×10^5^	0.2663
	SRB	(3.66±1.48)×10^2a^	(1.79±0.65)×10^2b^	(1.39±0.33)×10^2b^	0.0028

Note: values with different small letter superscripts in the same row mean that there was a significant difference (*P*<0.05), and the same small letter superscripts mean that there was no difference.

A The fecal sample replications in C group n = 10, D group n = 11, D+L group n = 11.

B The colon digesta sample replications in C group n = 6, D group n = 5, D+L group n = 6.

C The colon mucosa sample replications in C group n = 6, D group n = 5, D+L group n = 6.

### Oxidative Biomarkers and Antioxidant Enzymes in the Liver of Barrows

The data for liver oxidative biomarkers and antioxidant enzymes are summarized in Table 5. Hydrogen peroxide (H_2_O_2_) levels, inhibiting hydroxyl radical ability, NO concentrations, protein carbonyl, and MDA status were of no significant difference among the C, D, and D+L groups. However, significant effects were found on the total carbonyl and 8-OH-dG levels in the liver of barrows. Total carbonyl and 8-OH-dG levels in the D and D+L groups were significantly lower than in the C group, and no difference was found between D and D+L groups (Table 5). For the oxidant and anti-oxidant relative enzymes, only total-SOD, CuZn-SOD, and Mn-SOD were influenced by dietary treatment. The T-SOD levels in the D+L group were significantly higher than in the C and D groups. The CuZn-SOD enzyme activity in the D+L group was significantly higher than in the D group, and to have no difference compared to the C group. Finally, Mn-SOD activity in the D and D+L groups was significantly higher than in the C group. The CAT, GSH-px, GR, GSH, GSSH, and the ratio of GSH to GSSG were not influenced by dietary treatment (Table 5).

**Table 5:** Oxidative biomarkers and antioxidant enzymes status in the liver of barrows.

Items	Control (n = 6)	Daidzein (n = 5)	D+L (n = 6)	P
Total carbonyl (mg/g wt liver)	7.07±0.78^a^	4.69±1.10^b^	5.09±1.04^b^	0.002
Hydrogen Peroxide(H_2_O_2_) (mmol/g protein)	5.77±0.65	5.50±0.81	5.40±0.88	0.699
Inhibiting Hydroxyl Radical (U/mg protein)	25.15±6.34	26.19±3.47	30.42±6.52	0.280
NO (μmol/g protein)	0.41±0.04	0.32±0.05	0.36±0.07	0.079
Protein Carbonyl (nmol/mg protein)	5.16±0.73	4.24±0.80	4.40±0.44	0.076
8-OH-dG (ng/g wt liver)	337.00±47.43^a^	258.53±75.22^b^	244.54±26.67^b^	0.017
CAT (U/g protein)	78.34±10.54	78.34±9.68	84.23±8.93	0.509
Total-SOD (U/mg protein)	239.08±12.06^b^	251.43±15.11^b^	299.38±35.46^a^	0.002
CuZn-SOD (U/mg protein)	156.04±13.49^ab^	134.32±5.90^b^	169.73±29.03^a^	0.030
Mn-SOD (U/mg protein)	83.04±5.36^b^	117.11±15.13^a^	129.65±14.96^a^	<0.0001
MDA (nmol/mg protein)	2.62±0.51	2.27±0.59	2.11±0.30	0.174
GSH-px (activity unit)	20.08±1.56	21.70±2.13	20.49±20.70	0.417
GR (U/g protein)	7.34±2.07	5.81±1.33	6.17±1.50	0.310
GSH (μmol/g wt liver)	0.84±.09	0.81±0.10	0.77±0.07	0.383
GSSG (μmol/g wt liver)	0.17±0.03	0.17±0.02	0.19±0.02	0.300
GSH/GSSG	5.1±0.98	4.74±0.92	4.05±0.34	0.090

Note: values with different small letter superscripts in the same row mean that there was a significant difference (*P*<0.05), and the same small letter superscripts mean that there was no difference.

## Discussion

Several studies have attempted to manipulate equol production by supplementing prebiotics or probiotics with daidzein or soy isoflavones in humans and animals [27], [28]. Our previous studies showed that 1% (10g/L) of lactulose was able to improve equol production *in vitro* [13]. In the current study, lactulose showed clear promotion of equol production in the feces and urine of the barrows treated with daidzein.

Hydrogen, in particular, was found to stimulate equol production in an equol-producing mixed microbial culture from a human fecal sample [7]. Previous study investigated the interactions between methanogens, sulphate-reducing, and equol-producing bacteria under *in vitro* simulated intestinal conditions [8]. Results showed that supplementation equol-producing bacteria EPC4 significantly decreased the methanogenesis and sulphidogenesis in the faecal samples with methanogenic or sulphate-reducing abilities. Daidzein was also found to act as an electron acceptor in equol production that likely diverts hydrogen away from CH_4_ or H_2_S formation [8]. Our data suggests that lactulose may be a novel way of promoting equol production. Although the status of hydrogen gas in the colon was not measured, the isobutyric acid, isovaleric acid, branched chain fatty acid (BCFA), and total VFA levels in the colon digesta were improved by lactulose supplement, which has been reported the VFA levels in the colon digesta manipulated by lactulose corresponded with an increase of hydrogen gas production [29].

It is thought that certain bacteria in the intestinal microflora are greatly involved in equol bioconversion. Recently, bacteria that convert daidzein to equol were isolated from human, pig [7], [30]–[33] and rat [34], [35] feces or cecal contents. Although these equol-producing bacteria are different in individual and species, they play the comparable role in improving the greater efficacy of a soy food diet. So our dietary lactulose application to improve intestinal equol production in swine might be applied to human and other animals. To explore the intestinal microflora composition manipulation by lactulose in barrows treated with daidzein as well as the interactions between equol production and hydrogen-utilizing bacteria (methanogenic *Archaea* and sulfate-reducing bacteria), and due to the fact that equol formation largely occurs in the colon and distal intestine [36], the fecal, colon digesta, and mucosa samples were tested. Cluster results of DGGE profiles obtained from the colon digesta and mucosa showed that daidzein but not lactulose had selective pressure on the community of SRB and MPB, which is aggreed with previous study [8]. Moreover, our data also indicates that the hydrogen-producing prebiotic lactulose might shift the pathways of hydrogen utilization, thus changing the profiles of SRB in feces.

The real-time PCR results showed that the abundance of bacteroidetes and firmicutes in the feces were significantly higher in the D+L group than in the D and C groups. These findings are partly in agreement with our previous study [37], which reported that the fecal population of bacteroidetes in rates treated with daidzein and equol-producing bacterium ZX-7 had a strong correlation with fecal equol concentration. Lactulose was also found to have a reduction effect on the SRB population in the feces and colon mucosa. This result runs contrary to previous results [38] that reported that a strong equol producer phenotype correlated positively with the abundance of sulfate-reducing bacteria. The results from this study indicate a strong relationship between equol production and hydrogen-utilization bacteria. However, the relationship between equol-producing, hydrogen-utilizing, and the selected bacteria seems to be more complex than previously suggested [7], [8]. Further research is needed to explain the exact mechanism that lactulose serve as a hydrogen-producing prebiotic to enhance equol production *in vivo*.

It is well established that isoflavones have estrogenic and antioxidant capacities [3]. The conversion of daidzein to equol by intestinal microflora may be physiologically important, as equol has significantly greater antioxidant activity and estrogenic activity compared to daidzein [39]. In our experiment, dietary supplements with 1% lactulose significantly increased the T-SOD and CuZn-SOD activity in the liver of barrows treated with daidzein (Table 5). The antioxidant ability of pure equol in humans, cells, and rats is widely established [40], [41]. However, to our knowledge, no information is available on the effects of a hydrogen-producing prebiotic that increases equol production and improves antioxidants status *in vivo*. The effects of equol on oxidative stress and the antioxidant defense system in the livers of mice suggests that equol may act as an antioxidant through an inhibition of oxidative stress and the stimulation of catalase and SOD [40]. Considering our experimental term was only 22 days and the forming of equol in barrows’ intestinal was sustained and in a slow rate, and that the homeostasis of oxidative and antioxidant status is relatively stable in healthy pigs, it is not surprising that only T-SOD and CuZn-SOD activity in the liver were improved by lactulose manipulation. The results also indicated that using a hydrogen-producing prebiotic as an equol-promoting activator may be a novel way to improve the bioavailability of daidzein, which may be of benefit to broader populations of humans and animals.

In conclusion, we have shown that dietary supplemental lactulose can significantly improve equol-producing ability by changing microbial profile, the population and abundance of selected bacteria, especially the SRB population in the digesta and mucosa of colon. In addition, lactulose results in SCFAs profile shifting in the colon digesta. Furthermore, the addition of lactulose in the barrows’ diet improved the antioxidant status of the livers of barrows treated with daidzein, showing the potential of the hydrogen-producing prebiotic lactulose for enhancing equol production in animals and humans.

## Supporting Information

Table S1 **Formula composition and nutrient levels of animal diet.**
(DOCX)

Table S2 **Primer sequences used in this study.**
(DOCX)
