# Supplementary material for: Lactulose Increases Equol Production and Improves Liver Antioxidant Status in Barrows Treated with Daidzein
Source: PLoS One. 2014 Mar 25;9(3):e93163. doi: 10.1371/journal.pone.0093163 (PMC3965542; doi:10.1371/journal.pone.0093163)
Supplement: Table S1 — Formula composition and nutrient levels of animal diet. (DOCX) [file pone.0093163.s001.docx]

**Table S1 Formula composition and nutrient levels of animal diet**

| Ingredients | Control | Daidzein | D+L |
| --- | --- | --- | --- |
|  | g/100g diet | | |
| Corn | 66 | 66 | 66 |
| Corn protein powder | 10 | 10 | 10 |
| Casein | 4.3 | 4.3 | 4.3 |
| Fishmeal | 5 | 5 | 5 |
| Whey powder | 8 | 8 | 8 |
| sunflower oil | 1.5 | 1.5 | 1.5 |
| Lysine | 0.2 | 0.2 | 0.2 |
| Premix^a^ | 3 | 3 | 3 |
| Stone powder | 1.5 | 1.5 | 1.5 |
| Calcium hydrogen phosphate | 0.5 | 0.5 | 0.5 |
| Daidzein | - | 0.005 | 0.005 |
| Lactulose | - |  | 1 |
|  |  |  |  |
| Nutrient levels^b^ |  |  |  |
| DE（kcal/kg） | 3400 | 3400 | 3400 |
| CP(%） | 18.53 | 18.53 | 18.53 |
| EE(%) | 6.18 | 6.18 | 6.18 |
| CF(%) | 1.98 | 1.98 | 1.98 |
| Ca(%) | 1.25 | 1.25 | 1.25 |
| Total phosphorus (%) | 0.68 | 0.68 | 0.68 |

^a^Provided per kg of premix for weaned pigs: Fe 1800 mg; Cu 1020 mg; Zn 1800 mg; Mn 1200 mg; I 6 mg; Se 3.6 mg; VA 2200000 IU; VD 75000 IU; VE 150 mg; VK3 500 mg; VB1 500 mg; VB2 500 mg; VB6 3750 mg; VB12 750 mg; nicotinic acid 250 mg; calcium pantothenate 750 mg; folacin 250 mg; biotin 5 mg.

^b^The nutrient levels are calculated value.
