# Supplementary material for: Lactulose Increases Equol Production and Improves Liver Antioxidant Status in Barrows Treated with Daidzein
Source: PLoS One. 2014 Mar 25;9(3):e93163. doi: 10.1371/journal.pone.0093163 (PMC3965542; doi:10.1371/journal.pone.0093163)
Supplement: Table S2 — Primer sequences used in this study. (DOCX) [file pone.0093163.s002.docx]

**Table S2 Primer sequences used in this study**

| Primer | Sequence 5′−3′ | Reference |
| --- | --- | --- |
| U968-GC | CGCCCGGGGCGCGCCCCGGGCGGGGCGGGGGCACGGGGGGAACGCGAAGAACCTTAC | [1] |
| L1401 | CGGTGTGTACAAGACCC | [1] |
| 519f | CAGCCGCCGCGGTAA | [2] |
| 915rGC | CGCCCGCCGCGCCCCGCGCCCGGCCCGCCGCCCCCGCCCCGTGCTCCCCCGCCAATTCCT | [2] |
| AllBac296F | GAGAGGAAGGTCCCCCAC | [3] |
| Eub1369R | CCGRGAACGTATTCACCG | [4] |
| Firm934F | GGAGYATGTGGTTTAATTCGAAGCA | [5] |
| Firm1060R | AGCTGACGACAACCATGCAC | [5] |
| Bact1369 | CGGTGAATACGTTCYCGG | [4] |
| 1492R | GGWTACCTTGTTACGACTT | [4] |
| qmcrA-F | TTCGGTGGATCDCARAGRGC | [6] |
| qmcrA-R | GBARGTCGWAWCCGTAGAATCC | [6] |
| APS-FW | TGGCAGATMATGATYMACGGG | [7] |
| APS-RV | GGGCCGTAACCGTCCTTGAA | [7] |
| APS-RV-GC | CGCCCGCCGCGCCCCGCGCCCGGCCCGCCGCCCCCGCCCGGGGCCGTAACCGTCCTTGAA | [7] |
| T7 | TAATACGACTCACTATAGG | Promega |
| Sp6 | GATTTAGGTGACACTATAG | Promega |

**References**

1. Nubel U, Engelen B, Felske A, Snaidr J, Wieshuber A, et al. (1996) Sequence heterogeneities of genes encoding 16S rRNAs in Paenibacillus polymyxa detected by temperature gradient gel electrophoresis. J Bacteriol 178: 5636-5643.

2. Cheng Y, Mao S, Liu J, Zhu W (2009) Molecular diversity analysis of rumen methanogenic Archaea from goat in eastern China by DGGE methods using different primer pairs Lett Appl Microbiol 48: 585-592.

3. Layton A, McKay L, Williams D, Garrett V, Gentry R, et al. (2006) Development of Bacteroides 16S rRNA gene TaqMan-based real-time PCR assays for estimation of total, human, and bovine fecal pollution in water. Appl Environ Microbiol 72: 4214-4224.

4. Suzuki MT, Taylor LT, DeLong EF (2000) Quantitative analysis of small-subunit rRNA genes in mixed microbial populations via 5'-nuclease assays. Appl Environ Microbiol 66: 4605-4614.

5. Guo X, Xia X, Tang R, Zhou J, Zhao H, et al. (2008) Development of a real-time PCR method for Firmicutes and Bacteroidetes in faeces and its application to quantify intestinal population of obese and lean pigs. Lett Appl Microbiol 47: 367-373.

6. Denman SE, Tomkins NW, McSweeney CS (2007) Quantitation and diversity analysis of ruminal methanogenic populations in response to the antimethanogenic compound bromochloromethane. FEMS Microbiol Ecol 62: 313-322.

7. Deplancke B, Hristova KR, Oakley HA, McCracken VJ, Aminov R, et al. (2000) Molecular ecological analysis of the succession and diversity of sulfate-reducing bacteria in the mouse gastrointestinal tract. Appl Environ Microbiol 66: 2166-2174.
